# Combined Fertility Preservation Technique before Gonadotoxic Treatments in Cancer Patients

**DOI:** 10.22074/IJFS.2021.523540.1081

**Published:** 2021-10-16

**Authors:** Koray Görkem Saçıntı, Yavuz Emre Şükür, Murat Sönmezer, Cem Somer Atabekoğlu

**Affiliations:** Ankara University School of Medicine, Department of Obstetrics and Gynaecology, Ankara, Turkey

## Abstract

Although ovarian tissue cryopreservation is still considered as an experimental technique, several authors from around the world have reported successful and promising results. Currently, oocyte cryopreservation seems to be the most feasible technique for fertility preservation when there’s some kind of a time constraint in adolescents and adults. However, it has been estimated that a young woman would be expected to have a 94% likelihood of having a live birth with 20 mature frozen oocytes (1). At age 34 years, however, this expectation is decreased to 90% with 20 mature frozen oocytes. In addition to age-related limitations, an immediate obstacle for obtaining oocytes in cancer patients is the fact that only one controlled ovarian hyperstimulation (COH) cycle can usually be performed in these women because of time constraints, yielding a relatively low number of oocytes and/or embryos. For this reason, results from egg donation programs cannot be extrapolated to cancer patients, nor can the quality of oocytes be guaranteed. Hence, a combined fertility preservation technique can be of valuable in increasing the chances of successful future pregnancies following gonadotoxic cancer therapies. Previously, Dolmans et al. (2) suggested that cryopreservation of bilateral ovarian cortex followed by COH is a feasible and safe approach to preserve fertility before gonadotoxic treatment, and that the number of cryopreserved embryos was similar to the controls.

We have been offering the option of the combined technique to fertility preservation patients for a couple of years and have performed it in a series of eight candidate patients. All patients had enough time for COH before oncology treatments. We first performed laparoscopic ovarian resection for ovarian tissue cryopreservation and then started COH on postoperative day 0 or 1 in each patient (Table 1). The main point in our findings is that ovarian resection is performed from the side with less antral follicle count of the patients. We suggest that this approach can increase the oocyte yield in a single available COH cycle.

The data is limited on the effectiveness of combined technique and more long-term follow-up studies are needed in larger groups with appropriate controls. According to our clinical experience, we believe that combined technique is a valid approach, which is expanding beyond the experimental stage and has become a clinical technique for fertility preservation. We particularly suggest selecting the ovary with a low antral follicle count for wedge resection to increase oocyte yield. The information gathered from large international multicenter reports would encourage physicians to agree that the method should complete the experimental phase and be ready for wider clinical use in female fertility preservation.

**Table 1:** Data of the patients who chose combined technique for fertility preservation


No	Age (Y)	Diagnosis	AMH (ng/mL)	Right AFC	Left AFC	OTC	No. of oocytes collected	MII oocytes cryopreserved	Embryos cryopreserved

1	26	Breast cancer	4.5	6	8	Right	21	19	-
2	34	NHL	0.94	3	1	Left	5	5	-
3	37	Breast cancer	1.7	2	5	Right	13	8	-
4	25	Breast cancer	3.9	4	7	Right	16	15	-
5	15	Ewing sarcoma	0.4	3	1	Left	6	5	-
6	30	AML	2.4	7	5	Left	12	7	7
7	28	Rectum cancer	5.2	8	5	Left	18	13	7
8	37	Breast cancer	2.0	1	4	Right	12	7	3


NHL; Non-Hodgkin lymphoma, AML; Acute myeloid leukaemia, AMH; Anti-Müllerian hormone, AFC; Antral follicle count, OTC; Ovarian tissue cryopreservation, and MII; Metaphase II oocytes.
